# Enhanced genomic surveillance of enteroviruses reveals a surge in enterovirus D68 cases, the Johns Hopkins health system, Maryland, 2024

**DOI:** 10.1128/jcm.00469-25

**Published:** 2025-06-10

**Authors:** Amary Fall, Julie M. Norton, Omar Abdullah, Andrew Pekosz, Eili Klein, Heba H. Mostafa

**Affiliations:** 1 Division of Medical Microbiology, Department of Pathology, Johns Hopkins School of Medicine 1500 , Baltimore, Maryland, USA; 2 W. Harry Feinstone Department of Molecular Microbiology and Immunology, Johns Hopkins Bloomberg School of Public Health 25802 , Baltimore, Maryland, USA; 3 Department of Emergency Medicine, Johns Hopkins School of Medicine 1500 , Baltimore, Maryland, USA; 4 Center for Disease Dynamics Economics and Policy 439584, Washington, DC, USA; The University of North Carolina at Chapel Hill School of Medicine, Chapel Hill, North Carolina, USA

**Keywords:** EV-D68, enterovirus

## Abstract

This study reports increased Enterovirus D68 (EV-D68) circulation in 2024, re-establishing its biennial circulation cycle after its interruption during the COVID-19 pandemic. A total of 1,395 respiratory and cerebrospinal fluid (CSF) samples, positive for rhinovirus/enterovirus, collected from January to November 2024 were screened. EV-D68 was the predominant enterovirus detected (72.6% of EV-positive samples), with cases peaking in October, consistent with historical seasonal patterns. Demographically, children under 5 years were predominantly infected with EV-D68 (41.6% of cases). Phylogenetic analysis revealed the co-circulation of two EV-D68 subclades: B3 (71%) and A2 (29%). Subclade B3 was primarily associated with pediatric infections (median age: 5 years), while A2 was more common in adults (median age: 42 years). Comparative genomic analysis of the 2024 B3 genomes, along with genomes from 2018 and 2022, identified the emergence of four amino acid substitutions, including three in nonstructural proteins (3C: I597V; 3D: I950V, T2173A) and one in the structural protein VP2 (T145S). The six positive enterovirus CSF samples diagnosed in 2024 included six different types: EV-D68, E9, E30, E18, CV-A9, and CV-B1. Notably, the 2024 EV-D68 outbreak did not coincide with a reported increase in acute flaccid myelitis (AFM) cases. This study highlights the importance of EV-D68 genomic surveillance for monitoring EV-D68 evolution, given its association with severe respiratory disease and neurological complications. Enhanced surveillance is also critical for the early detection of the emergence of enteroviruses, such as EV-C105, identified in this study.

**IMPORTANCE:**

Enteroviruses (EVs), a genus within the *Picornaviridae* family, are small, single-stranded RNA viruses linked to a wide spectrum of diseases, including neurological conditions. Despite their prevalence, they remain understudied. EV-D68 and EV-A71 have raised global public health concerns due to outbreaks of acute flaccid myelitis (AFM) and encephalomyelitis in North America and Europe. EVs exhibit high genetic variability, and viral evolution has been associated with changes in neurovirulence. Notably, EV-D68 epidemics in 2014, 2016, and 2018 coincided with spikes in AFM cases. However, AFM reports from 2019 to 2022 were low, even with a significant increase in EV-D68 infections in 2022. We developed an EV-D68 genomic surveillance workflow to investigate genotype associations with severe disease. Our previous work linked amino acid substitutions in 2018 strains to increased disease severity. This study analyzes EV-D68 evolution in 2024 and documents the return of its biennial circulation pattern following disruption during the COVID-19 pandemic.

## INTRODUCTION

Human enteroviruses (EVs), members of the *Picornaviridae* family, are non-enveloped, single-stranded RNA viruses classified into four species (A–D) (1, 2). While EVs mainly cause mild or asymptomatic infections, certain genotypes are associated with severe respiratory disease and neurological complications, including acute flaccid myelitis (AFM), a polio-like paralysis that predominantly affects children (3–5). Among these, enterovirus D68 (EV-D68) has emerged as a critical public health concern due to its unique ability to cause biennial outbreaks of respiratory illness along with increased cases of AFM (4, 6).

EV-D68’s epidemiology has been characterized by biennial resurgences since the first global outbreak in 2014, with peaks in late summer and fall, mirroring patterns of rhinovirus transmission (7–9). However, the COVID-19 pandemic disrupted this cycle, reducing EV-D68 circulation in 2020–2021, likely due to non-pharmaceutical interventions and a possible alteration in population immunity (10–12). In 2022, EV-D68 re-emerged in the United States, but notably without the associated rise in AFM cases seen in previous outbreaks (2014, 2016, and 2018), raising questions about the contribution of viral evolution and host susceptibility (13–15).

A comparison of the 2018 and 2022 EV-D68 outbreaks revealed that subclade B3 remained the predominant circulating subclade. However, the 2022 strains exhibited increased genetic diversity and lacked the cluster of amino acid substitutions that was significantly associated with increased admissions in 2018 (13, 14). These findings highlighted the need for enhanced genomic surveillance to monitor emerging EV-D68 variants and their potential impact on disease severity and epidemiologic trends.

In this study, we report an increase in EV-D68 cases in Maryland in 2024 using an optimized surveillance protocol and screening a large number of positive rhinovirus/enterovirus samples. We also characterize the genotypes of enteroviruses associated with viral meningitis/encephalitis in 2024 and other circulating non-EV-D68 enteroviruses and rhinoviruses.

## MATERIALS AND METHODS

### Study population

Genomic enterovirus surveillance is routinely conducted on respiratory and CSF samples that test positive for rhinovirus/enterovirus (RV/EV) following the standard-of-care diagnosis at the Johns Hopkins Health System (JHHS) using the ePlex respiratory pathogen panels and an in-house laboratory developed test (LDT) (11, 13, 14). At our hospital, while routine laboratory diagnosis of symptomatic viral respiratory infections typically includes SARS-CoV-2, influenza, and RSV, extended respiratory panel testing that includes RV/EV is performed less frequently and is primarily recommended for hospitalized patients, immunocompromised individuals, and, to a lesser extent, pediatric and elderly patients (16). All available RV/EV-positive remnant samples, collected between January and November 2024, were used for enterovirus typing and whole genome sequencing. Clinical and demographic data were collected as previously described (11) through a bulk query of the electronic medical record system.

### RNA extraction, VP4/VP2 typing, and EV-D68 whole genome sequencing

Nucleic acid extraction was performed using the Chemagic 360 system (PerkinElmer) according to the manufacturer’s protocol, with elution in 60 µL. RV/EV samples were typed by amplifying the VP4/VP2 region using primers designed to amplify both virus species as previously described (16).

Library preparation was conducted using the Native Barcoding Kit (EXP-NBD196) and the NEBNext ARTIC Library Prep Kit, following the manufacturer’s instructions. Sequencing was performed on a GridION platform (Oxford Nanopore Technologies) using R10.4.1 flow cells.

FASTQ files generated from sequencing were analyzed using an in-house pipeline described previously (13). A comprehensive database of RV/EV reference genomes was used to identify the closest reference sequences. Draft genomes were assembled using the *mini_assemble* function within Pomoxis (version 0.3.15) and further refined to generate consensus sequences with Medaka Consensus (version 2.0.1). Genome quality, including sequencing depth, was assessed using *Samtools Depth* (version 1.21).

The types of VP4/VP2 sequences generated in this study were identified using the RIVM genotyping tool (http://www.rivm.nl/mpf/enterovirus/typingtool/).

Whole genome sequencing (WGS) was performed on samples that genotyped as EV-D68 using two overlapping amplicons as previously described (13). Sequencing and analysis of the FASTQ files followed the same pipeline described above for VP4/VP2 genotyping.

Complete genome sequences were aligned with reference genomes from GenBank using *Mafft* (version 7.450). Maximum likelihood trees for the complete genomes were carried out using IQ-TREE2 (version 2.0.6), with 1,000 bootstrap replicates. The ModelFinder, implemented in IQ-TREE2, was used to select the best-fitted nucleotide substitution model. Tree visualization was performed using an in-house script developed in the R programming language (https://www.R-project.org/). To identify and quantify the prevalence of amino acid substitutions in our 2024 B3 subclade sequences compared with our previous B3 subclade sequences from 2018 and 2022, we ran a Python script as previously described (13), using the Fermon strain as a reference.

## RESULTS

### Demographic and clinical characteristics of patients positive for enteroviruses

From January to November 2024, a total of 1,395 RV/EV-positive samples (total tested respiratory samples in this time frame ~17,454), including six CSF samples, were collected for VP4/VP2 sequencing. Of these, 84.5% (1,179/1,395) were successfully genotyped. Among them, 9.0% (106/1179) were identified as enteroviruses (EVs), while 91.0% (1,073/1,179) were classified as rhinoviruses (Table 1)

**TABLE 1:** Demographics and clinical characteristics of patients infected by EV-D68 and other enteroviruses at the Johns Hopkins Health System in 2024

	EV-D68	Other Enteroviruses	Total
N (%)	77 (72.6)	29 (27.4)	106 (100)
Female	41 (53.2)	13 (44.8)	54 (50.9)
Patient age			
0–5	32 (41.6)	15 (51.7)	47 (44.3)
6–17	14 (18.2)	9 (31)	23 (21.7)
18–44	12 (15.6)	2 (6.9)	14 (13.2)
45–64	10 (13.0)	1 (3.4)	11 (10.4)
65+	9 (11.7)	2 (6.9)	11 (10.4)
Race/ethnicity			
Black	33 (42.9)	9 (31)	42 (39.6)
Hispanic	6 (7.8)	2 (6.9)	8 (7.5)
Other	13 (19.9)	5 (17.2)	18 (17)
White	25 (32.5)	13 (44.8)	38 (35.8)
Comorbidities			
Asthma	18 (23.4)		18 (17)
Atrial fibrillation	4 (5.2)	1 (3.4)	5 (4.7)
Cancer	26 (33.8)	5 (17.2)	31 (29.2)
Cerebrovascular disease	11 (14.3)	1 (3.4)	12 (11.3)
Coronary artery disease	17 (22.1)	1 (3.4)	18 (17)
Diabetes	8 (10.4)		8 (7.5)
Heart failure	12 (15.6)		12 (11.3)
Hypertension	26 (33.8)	4 (13.8)	30 (28.3)
Immunosuppression	29 (37.7)	4 (13.8)	33 (31.1)
Kidney disease	16 (20.8)	3 (10.3)	19 (17.9)
Lung disease	25 (32.5)	8 (27.6)	33 (31.1)
Smoker	13 (16.9)	1 (3.4)	14 (13.2)
ED Visit	63 (81.8)	22 (75.9)	85 (80.2)
Co-infection^*a*^	7 (9.1)	5 (17.2)	12 (11.3)
Admitted	43 (55.8)	17 (58.6)	60 (56.6)
EV-related hospitalization	40 (51.9)	13 (44.8)	53 (50)
ICU-level of care	10 (13)	6 (20.7)	16 (15.1)
Supplemental oxygen	33 (42.9)	13 (44.8)	46 (43.4)

^*a*^ Co-infections detailed in Table S2.

**Fig 1:**
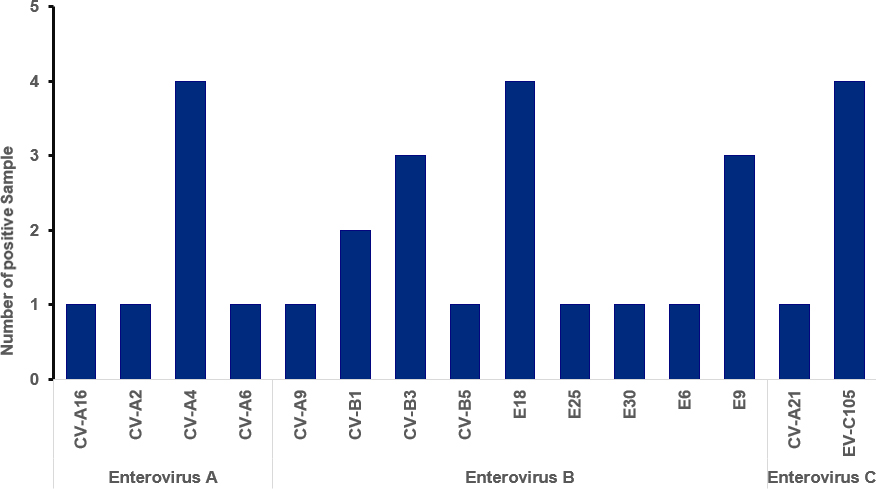
Genotype distribution of Enterovirus A, B, and C identified at the Johns Hopkins Health System from January to November 2024.

All four human enterovirus species (A–D) were detected, with EV-D being the most prevalent with a rate of 72.6% (77/106), followed by EV-B at 16.0% (17/106), EV-A at 6.6% (7/106), and EV-C at 4.7% (5/106). Notably, EV-D68 was the only EV-D subtype detected. Within the EV-B species, nine different genotypes were identified, with echovirus 18 (E18) being the most common (4/9), followed by echovirus 9 (E9, 3/9) and coxsackievirus B3 (3/9). The EV-A species was predominantly represented by coxsackievirus A4 (CV-A4) (4/7), while EV-C105 was detected in four of the five EV-C species (Fig. 1).

CSF-positive samples included one EV-D68 and five enterovirus B viruses, including E9, E18, E30, CV-A9, and CV-B1 (Table 2).

**TABLE 2:** Age range and clinical characteristics of patients with EV-positive CSF

Type	Age range (year)	Clinical presentation
EV-D68 (B3)	0–5	Fever
E9	0–5	Respiratory distress, meningitis
E30	0–5	Meningitis, fever
E18	0–5	Fever
CV-A9	18–44	Acute intractable headache, fever
CV-B1	0–5	Meningitis, acute intractable headache, fever, vomiting

Ages of patients infected with EV-D68 ranged from 1 month to 94 years with an average age of 19.8 and a median of 6 years. The majority of EV-D68 infections were in children under 5 years (41.6%), followed by adults older than 45 years (23.4%) and 6–17 years old (18.2%).

In terms of race and ethnicity, the highest number of EV-D68 cases was detected in Black individuals (42.9%), followed by White (32.5%) and Hispanic (7.8%) individuals. The most common comorbidities identified in the EV-D68 infected cohort were immunosuppression (37.7%), hypertension (33.8%), and lung disease (32.5%). Asthma was reported in 23.4% of EV-D68-infected patients. A large percentage of EV-D68 patients were admitted (55.8%), and 51.9% of admissions were EV infection-related. Additionally, 42.9% required supplemental oxygen, and 13% needed intensive care unit (ICU) level care. Outcomes, such as hospitalization, ICU admission, and the need for supplemental oxygen in EV-D68 infected cases, were not significantly different from those in non-EV-D68 enterovirus infections.

**Fig 2:**
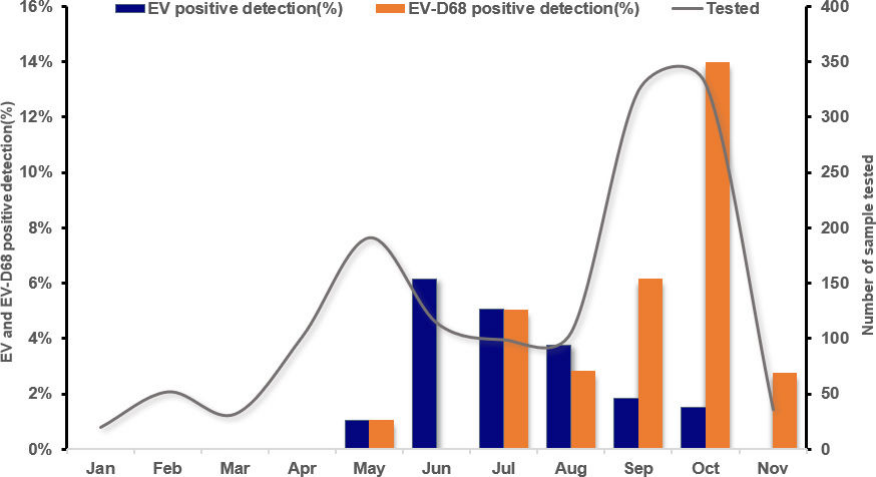
Circulation profile of Enterovirus D68 and other enteroviruses at the Johns Hopkins Health System from January to November 2024. EV-positive detection indicates all other non-EV-D68 EVs.

In 2024, EV-D68 was initially detected in May, with cases peaking in October (14.0%) (Fig. 2). In contrast, other enteroviruses were more frequently observed in June (6.1%) and July (5.1%).

### Phylogenetic analysis

**Fig 3:**
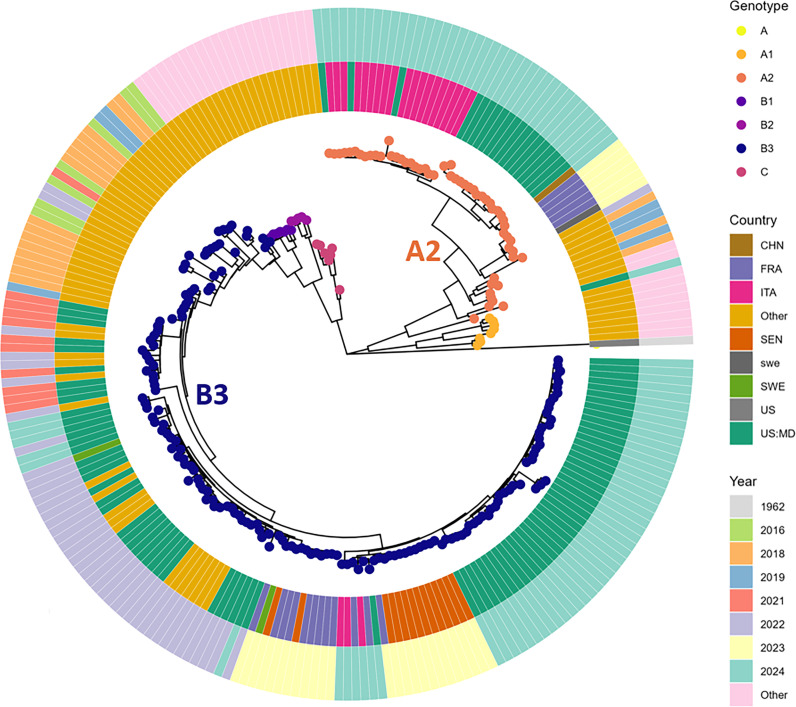
Phylogenetic relationships of EV-D68 strains identified from the Johns Hopkins Health system from 2024. We included complete genome sequences from other countries collected in 2023 and 2024 from GenBank. The phylogenetic tree was constructed using complete genomes, employing the maximum likelihood method in IQ-TREE2 with 1,000 bootstrap replicates and rooted by the Fermon strain.

**Fig 4:**
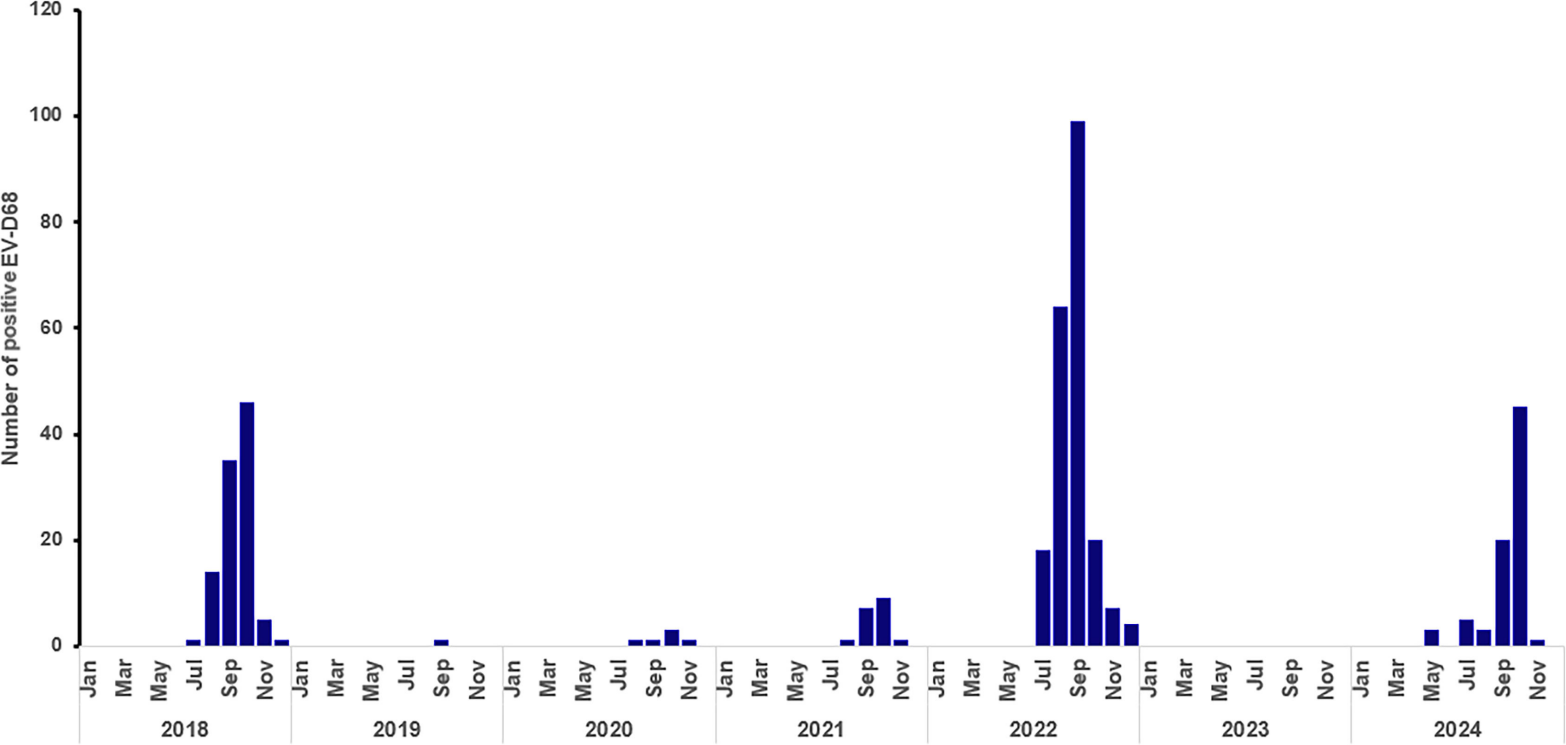
Enterovirus D68 cases identified from the Johns Hopkins Health system between 2018 and 2024.

Among the 77 EV-D68 samples processed for whole genome sequencing, complete genomes were successfully recovered for 70.1% (54/77), partial genomes (at least 50% coverage) were obtained in 19.5% (15/77), and sequencing failed in 10.4% (8/77) of the samples. Phylogenetic analysis of the complete and partial genome sequences (69/77) revealed the co-circulation of two subclades, B3 and A2, with subclade B3 predominating at 71.0% (49/69) of the sequences (Fig. 3). The 2024 EV-D68 subclade B3 genomes were divided into two distinct subclusters. The first, comprising a minority (6/49), defined by 25 nucleotide changes, and was most closely related to genomes characterized by our group in 2022. The predominant second subcluster (43/49) had 23 nucleotide changes and clustered with sequences from Senegal and France identified in 2023, as well as with genomes from Italy and France identified in 2024 (Table S1). Two subclusters were mainly observed within the A2 subclade. The predominant group (16/20) was defined by 17 nucleotide changes and was closely related to a genome from China characterized in 2024, whereas the second group (3/20) was defined by 31 nucleotide changes and clustered with genomes from Italy described in 2024 (Table S1). One strain did not cluster with either group but was instead closely related to a 2024 strain previously identified in Massachusetts (USA) based on BLAST analysis. Notably, the A2 genomes detected in Maryland in 2024 were closely related to sequences that circulated in France in 2023, although no A2 genomes were identified in Maryland in 2023, despite active surveillance (Fig. 4). Subclade A2 was predominantly detected in adult patients, with a median age of 42 years, whereas subclade B3 was primarily associated with pediatric infections, with a median age of 5 years. Multivariable logistic regression showed adults and elderly patients were more likely to be infected by the A2 subclade (odds ratio [OR], 7.28; 95% confidence interval [CI], 2.35–25.9).

In a comparative analysis of the 2024 EV-D68 subclade B3 whole genomes with genomes characterized in 2018 and 2022 (13), we identified four amino acid changes in over 10% of the 2024 genomes. Three of these substitutions were found in nonstructural proteins: one in 3C (I1597V (3C:I49V), present in 71% of the genomes) and two in 3D (I1950V (3D: I219V) in 74% and T2173A (3D: T442A) in 79%). The only substitution in a structural protein was T145S (VP2: T76S) in VP2, which was present in 24% of the sequences.

## DISCUSSION

In this study, we highlight a significant circulation of EV-D68 in Maryland in 2024 compared with 2023, indicating a return to the biennial circulation pattern (Fig. 4). We also show the predominance of subclade B3 and its similarity to 2022 genomes. The cluster of amino acid substitutions associated with a significant increase in admissions in 2018 remains undetected in 2024, similar to 2022. Likewise, the increase in cases in 2024 was not associated with an increase in EV-D68 virulence. Our study underscores the importance of genomic surveillance in monitoring the evolving epidemiology and genetic diversity of enteroviruses, including EV-D68.

The 2024 EV-D68 outbreak in Maryland exhibited a seasonal pattern, with cases peaking in October, consistent with the late summer and fall circulation historically observed for EV-D68 before the COVID-19 pandemic (7, 11, 13, 14, 17). The biennial pattern of EV-D68 activity appears to be returning after being temporarily disrupted during the COVID-19 pandemic, likely due to non-pharmaceutical interventions and potential shifts in population immunity (10–12).

Notably, we observed a decrease in the number of EV-D68 cases in 2024 compared with 2022. This decline may be attributed to the high genomic similarity between the strains circulating in both years, particularly within the VP1 region, which contains key antigenic sites. This suggests that a substantial portion of the population may have retained immunity from prior exposure, thereby reducing the pool of susceptible individuals in 2024 and limiting the virus’s ability to spread widely during that season.

Demographically, children under 5 years of age were the most affected group, accounting for 41.6% of EV-D68 cases. This finding is consistent with previous outbreaks, where young children have been disproportionately impacted due to their lack of prior immunity and higher susceptibility to severe respiratory illness (10, 18–20). Moreover, EV-D68 outbreaks often coincide with the start of the school year, when close contact among children facilitates viral spread. The median age in this study was 6 years, slightly higher than in our previous studies, where it was under 5 years (11, 13, 21). Notably, the median age observed here was lower than that reported in Italy in 2024 (22).

Interestingly, a higher proportion of EV-D68-infected individuals had a history of asthma (23.4%) compared with none among those infected with non-EV-D68 EVs. Previous studies reported that children with asthma are at increased risk of severe respiratory illness due to EV-D68 infection (4, 13, 20, 23).

Phylogenetic analysis revealed the co-circulation of two EV-D68 subclades, B3 and A2, with subclade B3 predominating, accounting for 71.0% of sequenced strains. The 2024 B3 genomes exhibited two distinct subclusters: one closely related to genomes from 2022, while the predominant subcluster showed genetic similarity to genomes from Senegal, France, Italy, and China in 2023–2024. Interestingly, the A2 genomes detected in the USA (Maryland), Italy, and China in 2024 were closely related to sequences that circulated in France in 2023. Notably, no A2 genomes were detected in Maryland in 2023, despite active surveillance. This temporal gap supports the notion of international seeding and underscores Maryland’s potential role as a sentinel site for early detection of globally emerging EVs.

The age-specific distribution of EV-D68 subclades A2 in adults (median age: 42 years) and B3 in children (median age: 5 years) likely reflects the interplay between immune maturation and viral evolution. These findings align with previous studies that have similarly highlighted age-related differences in viral subclade distribution (13, 18, 22, 24–26).

The significant association of subclade A2 with adults (OR, 7.28; 95% CI, 2.35–25.9) may result from the antigenic divergence of newer subclades from historical strains. Adults may lack robust immunity to these emerging variants, whereas prior exposure to older EV-D68 strains could confer partial protection against subclade B3 (27, 28). Conversely, pediatric populations, particularly infants older than 6 months, are immunologically naive as maternal antibodies wane, making them more susceptible to B3 and other recently circulating strains (28). However, the predominance of B3 and the lower prevalence of A2 in children suggest that additional factors, such as differences in viral fitness, transmission dynamics, or immune responses elicited by different subclades, may influence susceptibility.

The marked increase in pediatric EV-D68 infections post-2014 aligns with the global emergence of novel clades, including B3, which may evade pre-existing immunity in children (28). Furthermore, age-related differences in immune competence may also play a role, as children exhibit delayed adaptive immune activation and weaker mucosal defenses compared with adults (29), potentially facilitating infection with B3. However, the role of mucosal immunity in EV-D68 infection remains inadequately understood. Studies that investigate mucosal immune responses could inform the development of EV-D68 vaccines that could be similar to those successfully used for poliomyelitis (30).

These findings underscore the importance of age-specific immunity and viral adaptation in shaping EV-D68 epidemiology. The predominance of A2 in adults suggests that emerging subclades may exploit gaps in population immunity, while B3’s association with pediatric cases highlights the vulnerability of immunologically naive cohorts. Future studies should prioritize longitudinal serological surveys to track antigenic shifts and assess cross-protective immunity across age groups.

Our comparative genomic analysis of subclade B3 genomes from 2018, 2022, and 2024 identified four changes with frequencies above 10% in the 2024 genomes: T145S (VP2), I597V (3C), I1950V (3D), and A2173A (3D). Notably, none of the 2024 genomes had the cluster amino acid changes that were exclusively observed in our 2018 genomes, which we previously associated with increased EV-D68 admissions and the need for supplemental oxygen in 2018 (13). The absence of this cluster in both the 2022 and 2024 strains suggests that the genetic determinants of severe respiratory disease in EV-D68 may partially explain the differences observed between these seasons in the association with AFM.

Of particular interest, three of the substitutions identified in 2024 were located in nonstructural proteins: I597V in 3C protease, and I1950V and T2173A in 3D polymerase. These proteins are critical for viral replication and immune evasion. Although the specific functional implications of the I1950V substitution have not been well characterized, mutations in the 3C protease have been shown to affect viral polyprotein processing and the cleavage of host immune signaling molecules (31). Similarly, changes in the 3D polymerase can impact replication fidelity, adaptability, and viral fitness. The VP2:T76S substitution, found in 24% of 2024 sequences, is located in a capsid region that may affect virion stability or receptor interaction, though its functional significance remains unclear.

Importantly, none of the 2024 genomes contained previously reported neurovirulence-associated substitutions in the VP1 region (I553L, D554N, A650T, and K835E), which have been implicated in the development of AFM (32). This supports our hypothesis that genetic variation may contribute to the differing clinical presentations observed across seasons.

The detection of EV-D68 in CSF from a pediatric patient presenting with unspecified fever is an important finding in this study and is consistent with our previous report that identified EV-D68 CSF from patients with meningitis (33). The detection of EV-D68 in CSF is relatively rare, largely due to the transient nature of viremia and the typically low viral load within the central nervous system (CNS) (4). The infrequent detection may be attributed to several other factors, including the timing of CSF sample collection often occurring after the peak of viral replication. Nevertheless, the detection of EV-D68 in CSF underscores its potential to cause neurological complications and highlights the importance of considering EV-D68 in the differential diagnosis of febrile illnesses with suspected CNS involvement, particularly in pediatric populations during periods of increased EV-D68 circulation.

Our study also documents the emergence or re-emergence of EV-C105, consistent with recent reports from Europe (34–36). Although EV-C105 has historically been rarely reported, its detection in multiple geographic regions suggests it may be gaining epidemiological relevance. However, due to limited clinical data and the lack of a clear association with severe outcomes in our cohort, its clinical significance remains uncertain. Further genomic surveillance studies are needed to clarify its clinical impact and evolutionary dynamics.

The limitations of our study include the reliance on residual clinical samples, which may introduce selection bias, as samples from more severely ill patients or those with specific comorbidities could be overrepresented. Additionally, whole genome sequencing was not performed for other enteroviruses detected during the study period, limiting our ability to fully characterize their genetic diversity and potential clinical impact of novel subclades. Such work is in progress.

In conclusion, the 2024 EV-D68 outbreak in Maryland confirms the return of the biennial circulation pattern of EV-D68 following disruptions caused by the COVID-19 pandemic. The predominance of subclade B3, the detection of novel mutations, and the global interconnectedness of EV-D68 transmission emphasize the importance of enhanced genomic surveillance to monitor emerging variants and assess their potential impact on disease severity. These findings also highlight the need for continued surveillance of other enteroviruses to detect the emergence or re-emergence of viruses such as EV-C105.

EV-D68 was associated with severe disease and increased ICU admissions nationally in 2022 (14). However, enteroviruses currently lack structured surveillance systems in the US, and data linking genomic changes to clinical presentations and outcomes remain limited. Clinically, EV-D68 is often not differentiated, and the majority of the clinical laboratories are not capable of identifying it. Surveillance is limited, voluntary, or conducted as a part of research or epidemiological initiatives. Furthermore, viral etiologies of neurological disease may be undetectable in patients presenting later in the course of illness (37), and viruses are rarely detected in CSF in cases of AFM (38, 5). These challenges highlight the critical need to track respiratory transmission trends of EVs, monitor their evolution, and investigate correlations between viral genetic changes, clinical outcomes, and neurological disease. Future studies should aim to elucidate the functional consequences of the observed mutations and their potential roles in viral pathogenicity and transmissibility.

## Data Availability

Genomes were made publicly available in GenBank: PQ238722- PQ238727, PQ862937- PQ862967, PV624831-PV624847.

## SUPPLEMENTAL MATERIAL

Tables S1 and S2 jcm.00469-25-s0001.docx Table S1: Nucleotide changes that define B3 and A2 sub-clusters using the EV-D68 Fermon as Reference. Table S2. Respiratory virus co-infections in EV-D68 and non-EV-D68 Enterovirus-positive patients.

ASM does not own the copyrights to Supplemental Material that may be linked to, or accessed through, an article. The authors have granted ASM a non-exclusive, world-wide license to publish the Supplemental Material files. Please contact the corresponding author directly for reuse.

## References

[1] Knowles N, Hovi T, Hyypiä T, King A, Lindberg AM, Pallansch M, Palmenberg A, Simmonds P, Skern T, Stanway G. 2012. Family-picornaviridae, p 855–881. In Virus taxonomy: Ninth report of the International Committee on Taxonomy of Viruses. Elsevier, San Diego, CA.

[2] Simmonds P, Gorbalenya AE, Harvala H, Hovi T, Knowles NJ, Lindberg AM, Oberste MS, Palmenberg AC, Reuter G, Skern T, Tapparel C, Wolthers KC, Woo PCY, Zell R. 2020. Recommendations for the nomenclature of enteroviruses and rhinoviruses. Arch Virol 165:793–797. doi:10.1007/s00705-019-04520-6 31980941 PMC7024059

[3] Suresh S, Forgie S, Robinson J. 2018. Non-polio enterovirus detection with acute flaccid paralysis: a systematic review. J Med Virol 90:3–7. doi:10.1002/jmv.24933 28857219

[4] Elrick MJ, Pekosz A, Duggal P. 2021. Enterovirus D68 molecular and cellular biology and pathogenesis. J Biol Chem 296:100317. doi:10.1016/j.jbc.2021.100317 33484714 PMC7949111

[5] Messacar K, Asturias EJ, Hixon AM, Van Leer-Buter C, Niesters HGM, Tyler KL, Abzug MJ, Dominguez SR. 2018. Enterovirus D68 and acute flaccid myelitis-evaluating the evidence for causality. Lancet Infect Dis 18:e239–e247. doi:10.1016/S1473-3099(18)30094-X 29482893 PMC6778404

[6] Holm-Hansen CC, Midgley SE, Fischer TK. 2016. Global emergence of enterovirus D68: a systematic review. Lancet Infect Dis 16:e64–e75. doi:10.1016/S1473-3099(15)00543-5 26929196

[7] Messacar K, Pretty K, Reno S, Dominguez SR. 2019. Continued biennial circulation of enterovirus D68 in Colorado. J Clin Virol 113:24–26. doi:10.1016/j.jcv.2019.01.008 30825833

[8] Grizer CS, Messacar K, Mattapallil JJ. 2024. Enterovirus-D68 - a reemerging non-polio enterovirus that causes severe respiratory and neurological disease in children. Front Virol 4:1328457. doi:10.3389/fviro.2024.1328457 39246649 PMC11378966

[9] Monto AS. 2002. The seasonality of rhinovirus infections and its implications for clinical recognition. Clin Ther 24:1987–1997. doi:10.1016/s0149-2918(02)80093-5 12581541 PMC7133757

[10] Benschop KS, Albert J, Anton A, Andrés C, Aranzamendi M, Armannsdóttir B, Bailly J-L, Baldanti F, Baldvinsdóttir GE, Beard S, et al.. 2021. Re-emergence of enterovirus D68 in Europe after easing the COVID-19 lockdown, September 2021. Euro Surveill 26:2100998. doi:10.2807/1560-7917.ES.2021.26.45.2100998 34763750 PMC8646978

[11] Fall A, Gallagher N, Morris CP, Norton JM, Pekosz A, Klein E, Mostafa HH. 2022. Circulation of enterovirus D68 during period of increased influenza-like illness, Maryland, USA, 2021. Emerg Infect Dis 28:1525–1527. doi:10.3201/eid2807.212603 35642471 PMC9239864

[12] Park SW, Pons-Salort M, Messacar K, Cook C, Meyers L, Farrar J, Grenfell BT. 2021. Epidemiological dynamics of enterovirus D68 in the United States and implications for acute flaccid myelitis. Sci Transl Med 13:eabd2400. doi:10.1126/scitranslmed.abd2400 33692131

[13] Fall A, Abdullah O, Han L, Norton JM, Gallagher N, Forman M, Morris CP, Klein E, Mostafa HH. 2024. Enterovirus D68: genomic and clinical comparison of 2 seasons of increased viral circulation and discrepant incidence of acute flaccid myelitis—Maryland, USA. Open Forum Infect Dis 11:fae656. doi:10.1093/ofid/ofae656 PMC11575685 39564148

[14] Fall A, Han L, Abdullah O, Norton JM, Eldesouki RE, Forman M, Morris CP, Klein E, Mostafa HH. 2023. An increase in enterovirus D68 circulation and viral evolution during a period of increased influenza like illness, the Johns Hopkins health system, USA, 2022. J Clin Virol 160:105379. doi:10.1016/j.jcv.2023.105379 36652754

[15] Whitehouse ER, Lopez A, English R, Getachew H, Ng TFF, Emery B, Rogers S, Kidd S. 2024. Surveillance for acute flaccid myelitis - United States, 2018-2022. MMWR Morb Mortal Wkly Rep 73:70–76. doi:10.15585/mmwr.mm7304a1 38300829 PMC10843070

[16] Mostafa HH, Fall A, Norton JM, Sachithanandham J, Yunker M, Abdullah O, Hanlon A, Gluck L, Morris CP, Pekosz A, Klein EY. 2024. Respiratory virus disease and outcomes at a large academic medical center in the United States: a retrospective observational study of the early 2023/2024 respiratory viral season. Microbiol Spectr 12:e0111624. doi:10.1128/spectrum.01116-24 39162510 PMC11448398

[17] Gilrane VL, Zhuge J, Huang W, Nolan SM, Dhand A, Yin C, Salib C, Shakil F, Engel H, Fallon JT, Wang G. 2020. Biennial upsurge and molecular epidemiology of enterovirus D68 infection in New York, USA, 2014 to 2018. J Clin Microbiol 58:e00284-20. doi:10.1128/JCM.00284-20 32493783 PMC7448634

[18] Hodcroft EB, Dyrdak R, Andrés C, Egli A, Reist J, García Martínez de Artola D, Alcoba-Flórez J, Niesters HGM, Antón A, Poelman R, Reynders M, Wollants E, Neher RA, Albert J. 2022. Evolution, geographic spreading, and demographic distribution of enterovirus D68. PLoS Pathog 18:e1010515. doi:10.1371/journal.ppat.1010515 35639811 PMC9212145

[19] Ebada MA, Fayed N, Alkanj S, Allah AW. 2021. Enterovirus D-68 molecular virology, epidemiology, and treatment: an update and way forward. Infect Disord Drug Targets 21:320–327. doi:10.2174/1871526520666200715101230 32669078

[20] Fall A, Kenmoe S, Ebogo-Belobo JT, Mbaga DS, Bowo-Ngandji A, Foe-Essomba JR, Tchatchouang S, Amougou Atsama M, Yéngué JF, Kenfack-Momo R, et al.. 2022. Global prevalence and case fatality rate of Enterovirus D68 infections, a systematic review and meta-analysis. PLoS Negl Trop Dis 16:e0010073. doi:10.1371/journal.pntd.0010073 35134062 PMC8824346

[21] Fall A, Ndiaye N, Jallow MM, Barry MA, Touré CSB, Kebe O, Kiori DE, Sy S, Dia M, Goudiaby D, Ndiaye K, Niang MN, Dia N. 2019. Enterovirus D68 subclade B3 circulation in Senegal, 2016: detection from influenza-like illness and acute flaccid paralysis surveillance. Sci Rep 9:13881. doi:10.1038/s41598-019-50470-z 31554908 PMC6761155

[22] Pariani E, Piralla A, Pellegrinelli L, Giardina F, Porrello VN, Romano G, Galli C, Sandri L, Ferrari G, Binda S, Vezzosi L, Del Castillo G, Buoro S, Cereda D, Baldanti F, Respiratory viruses pandemic preparedness group Lombardy. 2024. Enhanced laboratory surveillance of respiratory infection disclosed the rapid rise of enterovirus D68 cases, northern Italy, August to September 2024. Euro Surveill 29:2400645. doi:10.2807/1560-7917.ES.2024.29.41.2400645 39392006 PMC11484921

[23] Midgley CM, Jackson MA, Selvarangan R, Turabelidze G, Obringer E, Johnson D, Giles BL, Patel A, Echols F, Oberste MS, Nix WA, Watson JT, Gerber SI. 2014. Severe respiratory illness associated with enterovirus D68 - Missouri and Illinois, 2014. MMWR Morb Mortal Wkly Rep 63:798–799. 25211545 PMC4584696

[24] Bal A, Sabatier M, Wirth T, Coste-Burel M, Lazrek M, Stefic K, Brengel-Pesce K, Morfin F, Lina B, Schuffenecker I, Josset L. 2019. Emergence of enterovirus D68 clade D1, France, August to November 2018. Euro Surveill 24:1800699. doi:10.2807/1560-7917.ES.2019.24.3.1800699 30670143 PMC6344839

[25] Böttcher S, Prifert C, Weißbrich B, Adams O, Aldabbagh S, Eis-Hübinger AM, Diedrich S. 2016. Detection of enterovirus D68 in patients hospitalised in three tertiary university hospitals in Germany, 2013 to 2014. Euro Surveill 21. doi:10.2807/1560-7917.ES.2016.21.19.30227 27195917

[26] Lau SKP, Yip CCY, Zhao PS-H, Chow W-N, To KKW, Wu AKL, Yuen K-Y, Woo PCY. 2016. Enterovirus D68 infections associated with severe respiratory illness in elderly patients and emergence of a novel clade in Hong Kong. Sci Rep 6:25147. doi:10.1038/srep25147 27121085 PMC4848506

[27] Harrison CJ, Weldon WC, Pahud BA, Jackson MA, Oberste MS, Selvarangan R. 2019. Neutralizing antibody against enterovirus D68 in children and adults before 2014 outbreak, Kansas City, Missouri, USA^1^. Emerg Infect Dis 25:585–588. doi:10.3201/eid2503.180960 30789123 PMC6390745

[28] Kamau E, Harvala H, Blomqvist S, Nguyen D, Horby P, Pebody R, Simmonds P. 2019. Increase in enterovirus D68 infections in young children, United Kingdom, 2006-2016. Emerg Infect Dis 25:1200–1203. doi:10.3201/eid2506.181759 30855226 PMC6537723

[29] Kollmann TR, Levy O, Montgomery RR, Goriely S. 2012. Innate immune function by toll-like receptors: distinct responses in newborns and the elderly. Immunity 37:771–783. doi:10.1016/j.immuni.2012.10.014 23159225 PMC3538030

[30] O’Grady M, Bruner PJ. 2025. Polio Vaccine, Treasure Island (FL). In StatPearls. StatPearls Publishing. 30252295

[31] Mukherjee A, Morosky SA, Delorme-Axford E, Dybdahl-Sissoko N, Oberste MS, Wang T, Coyne CB. 2011. The coxsackievirus B 3C protease cleaves MAVS and TRIF to attenuate host type I interferon and apoptotic signaling. PLoS Pathog 7:e1001311. doi:10.1371/journal.ppat.1001311 21436888 PMC3059221

[32] Leser JS, Frost JL, Wilson CJ, Rudy MJ, Clarke P, Tyler KL. 2024. VP1 is the primary determinant of neuropathogenesis in a mouse model of enterovirus D68 acute flaccid myelitis. J Virol 98:e0039724. doi:10.1128/jvi.00397-24 38869283 PMC11264684

[33] Fall A, Forman M, Morris CP, Gniazdowski V, Luo CH, Hanlon A, Miller H, Bergman Y, Mostafa HH. 2023. Enterovirus characterized from cerebrospinal fluid in a cohort from the Eastern United States. J Clin Virol 161:105401. doi:10.1016/j.jcv.2023.105401 36805602

[34] Fernandez-Garcia MD, Camacho J, Diez-Fuertes F, Ruiz de Pedro E, García-Ibañez N, Navascués A, Berengua C, Antequera-Rodriguez P, Ruiz-García M, Pastor-Fajardo MT, Cabrerizo M. 2025. Detections of rare enterovirus C105 linked to an emerging novel clade, Spain, 2019 to 2024. Euro Surveill 30:2500073. doi:10.2807/1560-7917.ES.2025.30.6.2500073 39949321 PMC11914964

[35] Nokhova AR, Saroyan TA, Solomatina MV, Gutova TA, Derko AA, Dubovitskiy NA, Murashkina TA, Sharshov KA, Shestopalov AM, Kurskaya OG. 2024. Genetic diversity and epidemiology of enteroviruses and rhinoviruses in children hospitalized with acute respiratory infections in Novosibirsk, Russia (2023-2024). Viruses 16:1924. doi:10.3390/v16121924 39772231 PMC11680272

[36] Pomari E, Malagò S, Ferrari G, Romano G, Mori A, Matucci A, Feletti R, Bonetti P, Baldanti F, Castilletti C, Piralla A. 2025. Unexpected pediatric cluster of enterovirus C105, Verona, Italy. Viruses 17:255. doi:10.3390/v17020255 40007009 PMC11861629

[37] Helfferich J, Neuteboom RF, de Lange MMA, Benschop KSM, Van Leer-Buter CC, Meijer A, Bakker DP, de Bie E, Braakman HMH, Brandsma R, Niks EH, Niermeijer J-M, Roelfsema V, Schoenmaker N, Sie LT, Niesters HG, Te Wierik MJM, Jacobs BC, Brouwer OF. 2023. Pediatric acute flaccid myelitis: evaluation of diagnostic criteria and differentiation from other causes of acute flaccid paralysis. Eur J Paediatr Neurol 44:28–36. doi:10.1016/j.ejpn.2023.03.002 36996587

[38] Messacar K, Spence-Davizon E, Osborne C, Press C, Schreiner TL, Martin J, Messer R, Maloney J, Burakoff A, Barnes M, Rogers S, Lopez AS, Routh J, Gerber SI, Oberste MS, Nix WA, Abzug MJ, Tyler KL, Herlihy R, Dominguez SR. 2020. Clinical characteristics of enterovirus A71 neurological disease during an outbreak in children in Colorado, USA, in 2018: an observational cohort study. Lancet Infect Dis 20:230–239. doi:10.1016/S1473-3099(19)30632-2 31859216 PMC11284833

