# Supplementary material for: Enhanced genomic surveillance of enteroviruses reveals a surge in enterovirus D68 cases, the Johns Hopkins health system, Maryland, 2024
Source: J Clin Microbiol. 2025 Jun 10;63(7):e00469-25. doi: 10.1128/jcm.00469-25 (PMC12239729; doi:10.1128/jcm.00469-25)
Supplement: Tables S1 and S2 — Table S1: Nucleotide changes that define B3 and A2 sub-clusters using the EV-D68 Fermon as Reference. Table S2. Respiratory virus co-infections in EV-D68 and non-EV-D68 Enterovirus-positive patients. [file jcm.00469-25-s0001.docx]

Table S1: Nucleotide changes that define B3 and A2 sub-clusters using the EV-D68 Fermon as Reference.

| **Sub-cluster** | **Nucleotides Defining the Sub-cluster** |
| --- | --- |
| **B3-SC1** | C345T (5UTR); C1477T (VP2); T2169C, C2277T, C2301T, C2328T and C2355T (VP3); T2661C, G2895A and A3189G (VP3); A3342G, G3579A and A3624G (2A); G5064A, T5118C, T5235A and A5286T (3A); A5454T, A5700G, G5829A and T5916A (3C); T6054C, T6192C, C6255T and T6792C (3D) |
| **B3-SC2** | C203T and T334C (5UTR); T1005C, T1008C, A1140G, T1195C (VP2); T1914C, C1939T, T2307C (VP3); A3546G, C3714T (2A); C4983T, T4989C (2C); T5214C (3A); T5418C, A5454C, G5478A, C5739T, C5859T (3C); G6660A, T6813C, T6835C, A6939G (3D) |
| **A2-SC1** | C168T (5UTR); A1329G and C1683T (VP2); C2259T (VP3); T2619C (VP1), G3984A (2B); G4257A, C4314T and C4638T (2C); T5169C (3A); G5790C and T5931C (3C); A6021G, A6333G, C6429T, T6952C and A6978G (3D) |
| **A2-SC2** | A2-2 : C98T, T318C, G624A, T641C, C642T, C644T and T645A ( 5UTR); C971T (VP2); C1708T, A2292T and A2325G (VP3); T2602C , A2682G, T3114C, A3132G and T3165C (VP1); C3324T, C3375T, T3435C, C3687T and A3734G(2A); G4086A and C4392T(2C); C5532T and C5790T(3C); C6429A, C6510T, T6543C, C6931T, G7248A and T7313C (3D). |

**Table S2**. Respiratory Virus Co-Infections in EV-D68 and Non-EV-D68 Enterovirus-Positive Patients

| ID | Enterovirus | Co-infection |
| --- | --- | --- |
| JHREV24385 | Enterovirus D68 | Parainfluenza 2 |
| JHREV24744 | Enterovirus D68 | Parainfluenza 2 |
| JHREV241009 | Enterovirus D68 | Parainfluenza 4 |
| JHREV241127 | Enterovirus D68 | Parainfluenza 1 |
| JHREV241338 | Enterovirus D68 | Adenovirus |
| JHREV241394 | Enterovirus D68 | RSV |
| JHREV241399 | Enterovirus D68 | Adenovirus |
| JHREV24227 | Enterovirus C | Metapneumovirus |
| JHREV24321 | Enterovirus B | Metapneumovirus |
| JHREV24605 | Enterovirus B | RSV |
| JHREV24912 | Enterovirus B | RSV |
| JHREV241171 | Enterovirus B | Coronavirus |
